# Isolation, screening, and optimization of the fermentation conditions for endophytic bacteria CHR2-1 of *Capparis hainanensis*

**DOI:** 10.3389/fmicb.2026.1693577

**Published:** 2026-03-13

**Authors:** Yanyan He, Fei Wang, Yu Zhao, Xian Xiao, Lin Yang, LanYing Wang

**Affiliations:** 1 School of Tropical Agriculture and Forestry, Hainan University, Haikou, China; 2 School of Ecology, Hainan University, Haikou, China

**Keywords:** endophytes, antagonistic activity, fermentation, identification of strains, CHR2-1

## Abstract

**Background:**

Banana wilt disease, caused by *F. oxysporum* f. sp. cubense (Foc), poses a severe threat to global banana production. Plant growth-promoting rhizobacteria (PGPR) offer eco-friendly alternatives to chemical fungicides for disease management.

**Methods:**

Surface-disinfected root, stem, and leaf tissues of *Capparis hainanensis* were used to isolate endophytic bacteria. Strain CHR2-1, exhibiting the strongest *in vitro* antagonistic activity against Foc, was selected. Antifungal mechanisms were evaluated using spore-germination inhibition assays and mycelial growth suppression assays. Greenhouse trials assessed the efficacy of disease control and growth promotion in banana seedlings. Optimal fermentation conditions were determined using response surface methodology. Phylogenetic analysis based on 16S ribosomal RNA (rRNA) gene sequencing identified the strain.

**Results:**

Channelrhodospin 1–2 (CHR2-1) suppressed 9/9 tested plant pathogens, with 88.4% inhibition against Foc. Spore germination inhibition exceeded 80%, while mycelial growth was reduced by 86.38%. In greenhouse trials, CHR2-1 reduced disease incidence by 65.62% (comparable to chemical fungicides) and increased banana-seedling biomass (height: +23.5%; root weight: +31.2%). Optimal fermentation parameters were 10.42 g/L peptone, 1.04 g/L sucrose, pH 7.5, 33 °C, and 180 rpm for 24 h. Phylogenetic analysis confirmed CHR2-1 as *Bacillus subtilis*.

**Conclusion:**

This study identifies *B. subtilis* CHR2-1 as a dual-function biocontrol agent with significant potential for sustainable management of banana wilt.

## 1 Introduction

With the increasingly severe problems of excessive pesticide residues in agricultural products, environmental pollution, and ecological imbalance caused by the overuse of chemical pesticides, green prevention and control technologies for plant diseases have become an important research direction for the sustainable development of global agriculture (Zhang et al., 2022). Biological control achieves disease control by using bioactive substances or the antagonistic effects between microorganisms, and offers advantages such as environmental friendliness and a low likelihood of drug resistance. In recent years, it has demonstrated high potential in agriculture and forestry. Among them, endophytes have become a focus of research due to their unique ecological functions. Studies have shown that endophytes can inhibit pathogenic bacteria and promote the growth of host plants through multiple mechanisms, such as secreting antibacterial substances (e.g., chitinase and β-1,3-glucanase), competing for ecological niches, inducing systemic resistance (ISR) in plants, and synthesizing plant hormones [e.g., indole-3-acetic acid (IAA) and gibberellin (gibberellic acid, GA)] (Ren et al., 2022). Three endophytes isolated from sponges by Abdulrahman were used to obtain extracts via solvent extraction, and these extracts exhibited significant antibacterial and antibiofilm activities (Smith et al., 2025). Pasakinskiene isolated three endophytic fungi from the roots of gramineous plants. By surface-sterilizing seeds and inoculating them with spore suspension, it was found that these endophytic fungi exerted a significant growth-promoting effect on *Hordeum vulgare* and Italian ryegrass, *Lolium multiflorum* (Pasakinskiene et al., 2024).

Endophytes form long-term mutualistic symbiotic relationships with their host plants, and their functional diversity far exceeds the traditional understanding. Studies have shown that by dynamically regulating the host’s metabolic network, endophytes play a core role in nutrient absorption (such as nitrogen fixation and phosphorus solubilization), stress response (such as drought resistance and salt tolerance) (Smith et al., 2025), and immune regulation. In terms of disease prevention and control, their mechanisms include the following: Direct antagonism: Secreting secondary metabolites (e.g., terpenoids and alkaloids) to damage the cell walls of pathogenic bacteria or inhibit the expression of virulence factors (e.g., chitinase degrading fungal cell walls) (Keshri et al., 2021); Ecological niche competition: Restricting the colonization of pathogenic bacteria by quickly occupying infection sites or consuming key nutrients (e.g., siderophores chelating iron elements) (Smith et al., 2025); Immune activation: Inducing plants to produce pathogenesis-related proteins (PR proteins) and reactive oxygen species (ROS) bursts, thereby enhancing induced systemic resistance (ISR) (Santos and Franco, 2023).

Beyond disease prevention and control, endophytes promote plant growth through multiple pathways: Nutrient efficiency enhancement: Synthesizing siderophores to improve iron absorption efficiency, or reducing ethylene levels via [1-aminocyclopropane-1-carboxylate deaminase (ACC)] deaminase to alleviate growth inhibition; Hormone regulation (Smith et al., 2025): Secreting indole-3-acetic acid (IAA) to promote the activity of root meristematic tissues and increase lateral root density as well as root hair development. For instance, the endophytic Bacillus strain SB001 enhances plant nutrient absorption through major facilitator superfamily (MFS) transporters; Metabolic synergy (Etesami and Glick, 2024): Promoting the host’s absorption of elements such as nitrogen and phosphorus (e.g., the phosphorus-solubilizing endophyte L21 activates soil phosphorus by secreting acid phosphatase) and improving stress resistance (e.g., salt-tolerant endophytes maintain cellular homeostasis by regulating proline metabolism) (Smith et al., 2025).

*Capparis hainanensis*, a medicinal plant endemic to southern China, represents an underexplored reservoir of beneficial endophytes. While species within the genus *Capparis* have traditionally been used for their pharmacological properties, research on their associated microbiomes remains limited. This study focuses on isolating and characterizing endophytic bacteria from *C. hainanensis*, with an emphasis on their potential for biocontrol and plant growth promotion (Nilavukkarasi et al., 2022; Li and Li, 2025).

*B. subtilisis* a well-documented biocontrol agent known for its ability to suppress soil-borne and foliar diseases through direct antagonism and induction of host resistance (Stuelke et al., 2023). Inhibition of Foliar Diseases: For diseases including tomato gray mold, cucumber downy mildew, and pepper phytophthora blight, *B. subtilis* activates the host’s defense response by inducing induced systemic resistance (ISR). Meanwhile, it secretes antibacterial proteins to interfere with the metabolism of pathogenic bacteria. For instance, the subtilin secreted by *B. subtilis* can inhibit the spore germination of Botrytis cinerea (the pathogen causing tomato gray mold) (Song et al., 2023). However, strains derived from unique ecological niches, such as *C. hainanensis*, may possess novel mechanisms or enhanced efficacy. In this work, we hypothesized that endophytic *B. subtilisis* from *C. hainanensis* exhibits broad-spectrum antagonistic activity against phytopathogens, coupled with plant growth-promoting traits. To test this hypothesis, we isolated endophytic bacteria from surface-sterilized *C. hainanensis* and screened them for antagonistic activity using dual-culture assays. Selected strains were further evaluated for their ability to inhibit spore germination and mycelial growth of target pathogens. Pot experiments were conducted to assess disease suppression and growth promotion in banana plants affected by Fusarium wilt disease. Additionally, fermentation conditions were optimized to enhance metabolite production, and strain identity was confirmed through phylogenetic analysis. This study aims to provide a theoretical foundation for developing novel biocontrol agents derived from endemic medicinal plants.

## 2 Materials and methods

### 2.1 Isolation, culture, and purification of endophytes from *Capparis hainanensis*

Fresh roots, stems, and leaves of the plant *C. hainanensis* from Yacheng County, Sanya City, Hainan Province, China (N 18°2343, E 109°1032) were first rinsed with tap water to remove the adhering soil and sand, and then dried indoors. The samples were disinfected by the conventional surface sterilization method: the plant samples were first immersed in 75% alcohol for 3–8 min (8 min for roots and stems, 3 min for leaves), then rinsed with sterile water 4 times, and then immersed in 0.1% mercuric chloride for 2–3 min. After that, they were rinsed with sterile water 5 times. Finally, the samples were pressed into potato dextrose agar (PDA, the medium components are 200 g of potato, 20 g of glucose, 18 g of agar, and distilled water to a final volume of 1,000 mL), NA (the medium components are 3 g of beef extract, 10 g of peptone, 5 g of sodium chloride, 18 g of agar, and distilled water to a final volume of 1,000 mL, pH 7.2), and Gao’s No. 1 agar plate (Guangdong Huankai Microbial Sci. & Tech. Co., Ltd.) (the medium components are 20 g of soluble starch, 1 g of potassium nitrate, 0.5 g of potassium dihydrogen phosphate, 0.5 g of sodium chloride, 0.5 g of magnesium sulfate heptahydrate, 0.01 g of ferrous sulfate heptahydrate, 15 g of agar powder, and 1,000 mL of distilled water, pH 7.2–7.4) for 15 min and then removed. The epidermis of the samples was removed with a scalpel. One part was cut into thin slices of approximately 5 × 5 mm and placed on the fungal isolation medium (PDA) for culture at 25–28 °C under constant temperature for 3–5 days. The other part was crushed and ground for 20 min, and a small amount of the grinding liquid was spread onto the bacterial isolation plate (nutrient agar [NA]) and the actinomycetes isolation plate (Gao’s No. 1) for constant-temperature culture at 25 °C for 24–48 h. The plates with pressed tissue blocks were normally cultured as control 1 to detect whether the surface disinfection was thorough. The final rinse water from the samples was spread onto PDA, NA, and Gao’s No. 1 plates (Guangdong Huankai Microbial Sci. & Tech. Co., Ltd.), respectively, and was normally cultured as control 2 to assess whether the surface disinfection was thorough. The endophytic fungi were purified by the tip culture method, and the endophytic bacteria and actinomycetes were purified by the streaking method. The purified strains were, respectively, preserved on PDA, NA, and Gao’s No. 1 slants for future use (Chaudhary et al., 2024).

### 2.2 Antagonistic activity of endophytic bacteria

Antagonistic activity of endophytic fungi was adopted by the confrontation culture method. The test strains are as follows: *F. oxysporum* f. sp. cubense (Foc) race 4 (FO, ATCC 76255), *F. oxysporum* f. sp. Niveum (FO, ATCC 18467), *Rhizoctonia solani* (RS, ATCC 58938), *Colletotrichum musae* (CM, ATCC 96167), *Colletotrichum gloeosporioides* (Penz.) (CG, ATCC 16330), *Pestalotia palmarum* Cooke (PP, ATCC 22386), *Corynespora cassiicola* (CC, ATCC 36294), *Botryodiplodia theobromae* (BT, ATCC 10936). At 25 °C, the target fungal cakes (ɸ = 4 mm) were inoculated in the center of a PDA plate. Using an inoculation ring, two stripes of endophytic bacteria (adjusted to a final concentration of 1 × 10^6^ spores/mL using a spectrophotometer) were created approximately 2 cm from the target fungi. The blank control (Dc) had no endophytic stripes. The number of biological replicates for each treatment group was *n* = 3. The strain (Dt) was cultured at 28 °C for 3 days, and all experiments were repeated 3 times. The diameter of fungal colonies was measured using the cross method, and the inhibition rate was calculated using the following formula.


$$\text{Inhibiting rate}\;(\%)=\frac{\mathrm{Dc}-\mathrm{Dt}}{\mathrm{Dc}-4}\times 100$$

### 2.3 Identification of endophytic bacteria CHR2-1

Endophytic bacteria CHR2-1 were identified by morphological, biochemical, and physiological characteristics. Morphological features include colony morphology, Gram staining, and flagella staining. D-glucose, D-xylose, L-arabinose, and D-mannitol were determined with seven different sugars as carbon sources. Determination of catalase, lecithinase, urease, aerobic action, injection of acetic acid, phenylalanine oxidative deamination, litmus milk and purple phosphorus reaction; starch hydrolysis, gluten liquefaction, methyl red and hydrogen sulfide production, sodium chloride and propionate utilization. The 16S ribosomal DNA (rDNA) gene was amplified by PCR (ABI company, United States) with Taq DNA polymerase and the conserved primers F (5′-AGAGTTTGATCCTGGCTCAG-3′) and R (5′-GGTTACCTTGTTACGACTT-3′) (Smith et al., 2025). The conditions for thermal cycling were as follows: denaturation of the target DNA at 94 °C for 5 min, followed by 35 cycles at 94 °C for 30 s, primer annealing at 55 °C for 35 s, and DNA elongation at 72 °C for 1 min. At the end of the cycling, the reaction mixture was held at 72 °C for 8 min. Polymerase chain reaction (PCR) amplification was detected by agarose gel electrophoresis in Shanghai Yingjun Biotechnology Co., Ltd. The PCR product obtained was sequenced by an automated sequencer. The sequence was compared for similarity to bacterial reference species in the GenBank database using Basic Local Alignment Search Tool (NCBI BLAST); available at http://www.ncbi.nlm.nih.gov/BLAST/. The phylogenetic tree was constructed using the neighbor-joining method in MEGA5.10 software (Feng et al., 2023).

### 2.4 Determination of endophytic bacteria CHR2-1 on the spore germination

According to Fang Zhongda’s method, the spore suspension of the target fungus was prepared. Target fungi were inoculated onto PDA medium for 5 days under 28 °C, and then eluted with 0.1% Tweens-80 (Beijing Labgic Technology Co., Ltd.) sterile water. A spore suspension was prepared at 1 × 10^5^ colony-forming unit (cfu)/mL.

A small amount of sterile water was added into the endophytic bacteria CHR2-1 to uniform bacteria suspension, and 50 mL bacteria suspension was inoculated in the Lysogeny Broth (LB) culture solution (yeast extract 5.0 g, peptone 10.0 g, sodium chloride 10.0 g, distilled water 975 mL, pH 7.2 ~ 7.4) and NA culture solution (beef extract 3 g, peptone 10.0 g, sodium chloride 5.0 g, distilled water 982 mL, pH 7.2 ~ 7.4), respectively, shaking with a speed 180 r/min and cultivating for 24 h under the temperature of 28 °C. The above solution was centrifuged for 10 min at a speed of 3,000 r/min, and the supernatant was filtered with a 0.2 μm bio-filter. A 0.5 mL filtrate and a 0.5 mL spore suspension were transferred into microscope concave slides and mixed to cultivate for 8 ~ 12 h at 28 °C. Spore suspension was replaced with 0.1% Tweens-80 (Beijing Labgic Technology Co., Ltd.) sterile water as the blank control; all experiments were performed in triplicate. Five fields of view were observed randomly for each concave slide to count spore germination; the total number of spores should be not less than 300. The spore germination rate (SGR) and Spore germination inhibiting rate (SGIR) were calculated using the following formula (Mondol et al., 2016).


$$\mathrm{SGR}\;(\%)=\frac{\text{Number of germinated spores}}{\text{Total number of spores}}\times 100$$

$$\text{SGIR}\;(\%)=\frac{\text{SGRck}-\text{SGRtr}}{\text{SGRck}}\times 100$$

SGRck: spore germination rate of control group; SGRtr: spore germination rate of treatment group.

### 2.5 Pot experiment of endophytic bacteria CHR2-1 against banana wilt disease

*F. oxysporum* was inoculated in the Czapek medium for 7 days at room temperature 25 °C, and then diluted to 10^7^ cfu/mL with sterile water. Non-banana plantation soils were collected from the experimental field in Hainan University, passed through the 20-mesh sieve, sterilized at 160 °C for 2 h, and then put into flowerpots with a diameter of 30 cm. The roots of banana seedlings with four leaves were cleaned with clean water. The banana seedlings were planted in flowerpots. After 14 days, the root of each banana seedling was irrigated with 50 mL of endophytic bacteria CHR2-1 suspension (10^9^ cfu/mL); each treatment was 10 banana seedlings. The blank control was sterile water, and the fungicide control was 95% hymexazol DP (1,200-fold). After cultivation for 4 days, the root was injured and inoculated with 20 mL *F. oxysporum* culture solution. All experiments were performed in triplicate, all potted plants in the treatment groups were arranged in the greenhouse using a completely randomized design or a randomized block design, and the location was changed once a week to minimize the impact of microenvironment differences. After 14, 22, 30 days, perform one-way analysis of variance on different indicators (such as plant height, disease index, and fresh weight), the disease index and the control effect were calculated according to the following formula, respectively (Shen et al., 2015).

The grading standards of *F. oxysporum* f. sp. cubense can be modified with reference:

Grade 0: Healthy plant;

Grade 1: The leaves of the lower part of the plant are withered;

Grade 2: 0–20% of the leaves are withered;

Grade 3: 20–40% of the leaves are withered;

Grade 4: 40–60% of the leaves are withered;

Grade 5: 60–80% of the leaves are withered;

Grade 6: The entire plant is withered and dead.


$$\text{Disease Index}=\frac{\sum (\begin{array}{l} \text{Number of diseased plants of each grade}\times \\ \text{value of relative grade} \end{array})}{\text{Total number inspected}\times 6}\times 100$$

$$\text{Control Effect}\;(\%)=\frac{\begin{array}{l} \text{Sterile water controlled disease index}-\\ \text{Treated disease index} \end{array}}{\text{Sterile water controlled disease index}}\times 100$$

### 2.6 Determination of the growth-promoting effect of endophytic bacteria CHR2-1 on banana seedlings

When the banana seedlings were transplanted after 15 days, the height (overground parts) and the stem diameter (3 mm off the ground soil) of the banana seedlings were measured. After 45 days, when 50 mL of endophytic bacteria CHR2-1 suspension (10^9^ cfu/mL) was applied, the height and stem diameter of the banana plants were measured, and the increment value of the height and stem diameter was counted. At the same time, banana plants were removed, washed with clear water, and then cut into the overground and underground portions. The fresh weight was calculated of the overground part and the underground part.

### 2.7 Optimization of the culture conditions of endophytic bacteria CHR2-1

Endophytic bacteria CHR2-1 were inoculated in LB culture solution and shaken for 24 h with a rotation speed of 180 r/min at 28 °C, and served as the seed solution to use.

#### 2.7.1 Optimization of culture temperature

Five milliliter seed solution was inoculated in the 95 mL LB culture solution, which was shaken and cultivated for 24 h with a rotation speed of 180 r/min at 23 °C, 28 °C, 33 °C, 38 °C, and 43 °C, respectively. The optical density (OD) value was measured at 630 nm using a spectrophotometer.

#### 2.7.2 Optimization of carbon source

Glucose, maltose, sucrose, fructose, and mannose were looked at as the external carbon source. The external carbon source (5 g/L) and 5 mL seed solution were inoculated into 90 mL LB culture solution, and the culture was then cultivated for 24 h with a rotation speed of 180 r/min at 33 °C, and no external carbon source was used as the blank control. OD was measured at 630 nm using a spectrophotometer.

#### 2.7.3 Optimization of nitrogen source

The yeast powder was replaced by other nitrogen sources, such as KNO_3_, (NH_4_)2SO_4,_ and beef extract, to prepare the LB culture solution. Five milliliters of seed solution was inoculated into the 95 mL modified LB culture solution. The culture conditions and measurement method were the same as those described above.

#### 2.7.4 Optimization of the pH value

On the basis of optimization of the carbon source and nitrogen source, the pH value of the culture solution was adjusted to 4.0, 4.5, 5.0, 5.5, 6.0, 6.5, 7.0, 7.5, 8.0, 8.5, and 9.0 with 1 mol/L HCl or NaOH, respectively, which was sterilized for 20 min at 121 °C. Whereafter, the pH value of the culture solution was calibrated with the sterilized 1 mol/L HCl or NaOH. The seed solution was inoculated into the culture solution of different pH values. The culture conditions and measurement method were the same as those described above.

### 2.8 Optimization of the fermentation conditions of endophytic bacteria CHR2-1

LB culture solution was modified on the basis of the culture conditions, peptone, potassium nitrate, sodium chloride, and sucrose were used as four factors (Table 1), and the quadratic general rotary unitized design method was used to optimize the fermentation conditions of endophytic bacteria CHR2-1. Each factor was set to 5 levels, ∆j was the space of each factor shown in Table 1.

**Table 1:** The design of the four factors and the five levels.

Level	*x*_1_ peptone (g/L)	*x*_2_ sucrose (g/L)	*x*_3_ potassium nitrate (g/L)	*x*_4_ sodium chloride (g/L)
2	10	5	5	10
1	7.75	3.875	3.875	7.75
0	5.5	2.75	2.75	5.5
−1	3.25	1.625	1.625	3.25
−2	1.0	0.5	0.5	1.0
∆j	2.25	1.125	1.125	2.25

Endophytic Bacteria CHR2-1 seed culture solution (1 mL) was inoculated in 19 mL PDA culture medium, shaken for cultivation for 3 days with a rotation speed of 180 r/min at 33 °C, and then filtered with a bio-filter. FO (ATCC 76255), inoculated onto PDA culture medium, was washed with sterile water to prepare the spore suspension. The spore suspension (200 μL) was transferred to the PDA plate (ɸ = 12 cm). Then, four sterilized Oxford cups were put on the PDA plate. Filtrate of CHR2-1 (200 μL) was added into each Oxford cup, which was cultured at 25 °C for 3 days. The diameter of the inhibition zone was measured with the crossing method.

### 2.9 Statistical analysis

All data were analyzed by ANOVA and expressed as mean ±SD. Analysis of variance and differences between samples were assessed using Duncan’s multiple range test, with *p* < 0.05 considered statistically significant.

## 3 Results and discussion

### 3.1 Isolation and antagonistic activity of endophytic bacteria

Fifteen strains of endophytic bacteria were isolated from the roots (eight strains), stems (seven strains), and leaves (none) of the *C. hainanensis* by the surface disinfection method. Only six strains of endophytic bacteria showed good antagonistic activities against the target fungi by the confrontation method (Table 2).

**Table 2:** Result of antagonistic activity of endophytic bacteria (inhibiting rate %).

Target fungi	CHR2-1	CHR2-4	CHR2-7	CHR2-8	CHS2-1	CHS2-6
FO (ATCC 76255)	88.40 ± 0.04a	73.20 ± 0.06b	77.46 ± 0.07b	65.28 ± 0.02c	67.61 ± 0.08c	50.13 ± 0.05d
FO (ATCC 18467)	66.90 ± 0.06a	55.93 ± 0.05b	45.81 ± 0.03c	43.24 ± 0.07cd	23.93 ± 0.12e	43.21 ± 0.09cd
RS (ATCC 58938)	75.73 ± 0.03a	38.36 ± 0.06e	67.43 ± 0.08b	62.10 ± 0.02c	51.53 ± 0.05d	35.75 ± 0.06e
CM (ATCC 96167)	75.50 ± 0.07a	56.70 ± 0.08bc	56.31 ± 0.02bc	73.21 ± 0.06ab	33.12 ± 0.04e	46.23 ± 0.09d
CG (ATCC 16330)	64.44 ± 0.14a	44.52 ± 0.08c	66.41 ± 0.02a	55.35 ± 0.05b	4.60 ± 0.08f	42.73 ± 0.07cd
PP (ATCC 22386)	53.71 ± 0.05a	47.54 ± 0.11ab	43.76 ± 0.07bc	28.93 ± 0.03e	34.21 ± 0.08d	16.15 ± 0.10f
CC (ATCC 36294)	82.02 ± 0.06a	55.73 ± 0.08d	78.43 ± 0.04ab	47.14 ± 0.07e	32.32 ± 0.09f	76.11 ± 0.05b
BT (ATCC 10936)	77.54 ± 0.03a	56.51 ± 0.06b	53.25 ± 0.02bc	42.13 ± 0.08d	23.61 ± 0.15e	56.24 ± 0.08b

Values within the same row followed by different lowercase letters (a–f) are significantly different according to Tukey’s HSD test (*p* < 0.05). Data are presented as mean ± standard deviation (*n* = 3).

**Figure 1:**
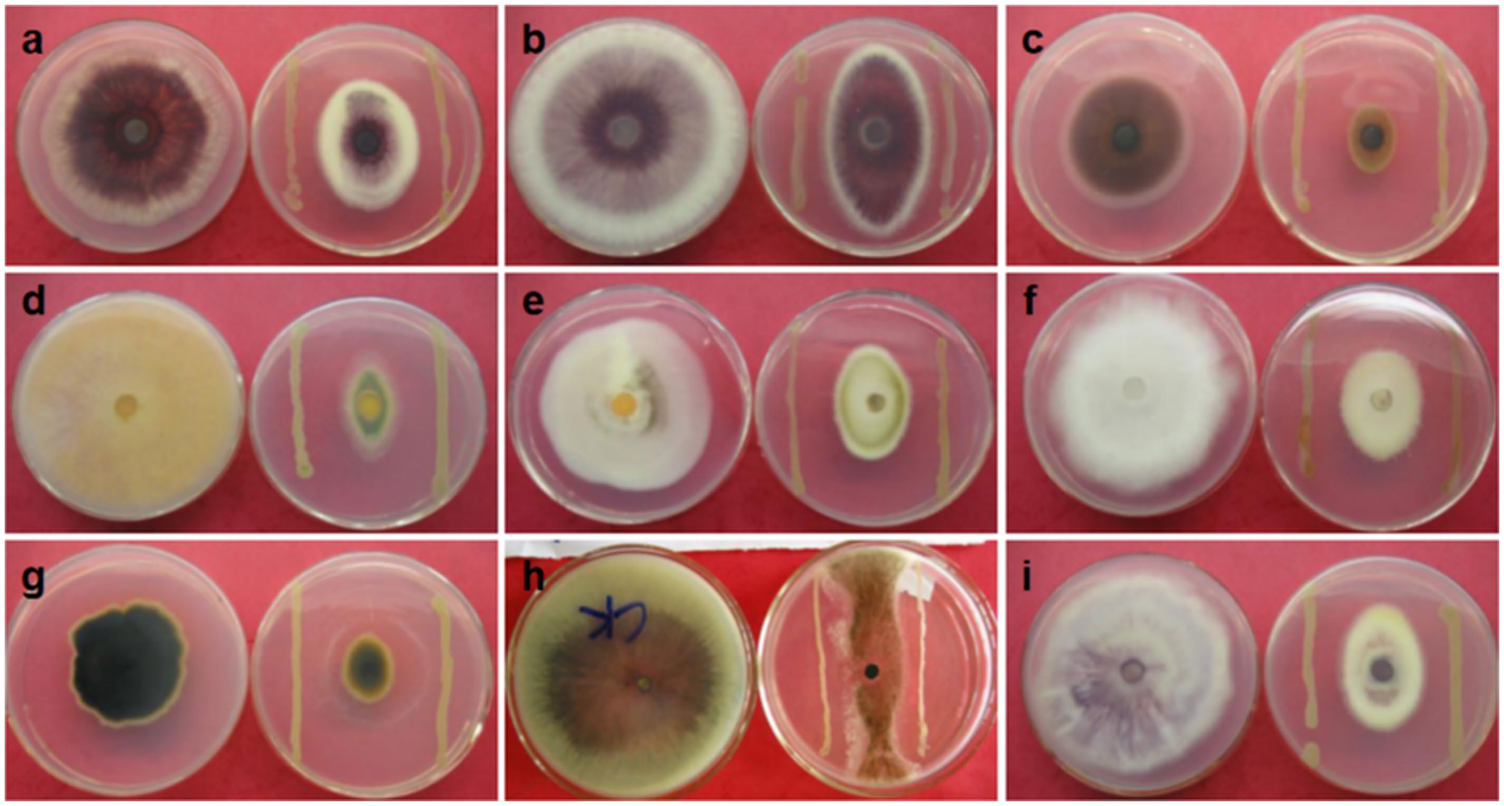
Antagonistic activity of endophytic bacterium CHR2-1. **(a)** *Fusarium oxysporum* f. sp. *cubense* strain, **(b)** *Fusarium oxysporum* f. sp. *melonis* strain, **(c)** *Rhizoctonia solani* strain, **(d)** *Colletotrichum gloeosporioides* strain, **(e)** *Colletotrichum acutatum*, **(f)** *Pestalotiopsis palmarum* strain, **(g)** *Corynespora cassiicola* strain (rubber tree leaf fall pathogen), **(h)** *Phomopsis mangiferae* strain, **(i)** *Periconia heveae* strain.

Table 2 showed that CHR2-1 isolated from the roots of *C. hainanensis* exhibited excellent broad-spectrum antagonistic activities against the 8 target fungi, and its inhibiting rates were all more than 50%, especially its rate was up to 88.40% against FO (ATCC 76255); second, its rate was up to 82.02% against CC (ATCC 36294). In addition, CHR2-4 and CHR2-7 isolated from the root of *C. hainanensis* also had good antagonistic activities. Moreover, FO (ATCC 76255) was the most sensitive by comparison with the target fungi; the second was CC (ATCC 36294), CM (ATCC 96167), and RS (ATCC 58938). As antagonistic activities and broad-spectrum activities of endophytic bacteria CHR2-1 were the best, endophytic bacteria CHR2-1 were selected to carry out the following research (Figure 1).

### 3.2 Identification of endophytic bacteria CHR2-1

**Figure 2:**
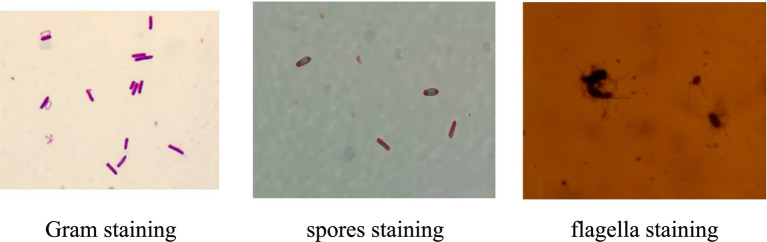
CHR2-1 staining of the strain.

After being inoculated for 24 h in a beef extract peptone plate by streak plate technique, the strain CHR2-1 displayed a flat, opaque, and white colony. The colony’s surface was rough and dry. Gram staining, spores staining, and flagella staining of the strain indicated this stain was a Gram-positive bacteria, rod-shaped types, and had central spores and peri-flagella (Figure 2).

The physiological and biochemical characteristics of endogenous bacterial CHR2-1 showed that the bacterial strain produced catalase and contact enzyme, but did not produce lecithinase, urease, and indole acetic acid. The strain was aerobic and could grow in the culture medium at pH 5.7 and in medium containing 2–7% NaCl. The strain could liquefy the gelatin, hydrolyze starch, but could not change the litmus milk color nor oxidize and deaminate to phenylalanine, nor decompose the sulfur-containing amino acid in the culture medium to hydrogen sulfide. The methyl red and Voges–Proskauer (VP) reactions of the strain were positive. The strain did not utilize propionate but grew well in the culture medium containing glucose, sucrose, xylose, fructose, and other carbon sources as the sole carbon source. The strain was fermented D-glucose, L-arabinose, D-xylose, and D-mannitol to produce acid (Table 3).

**Table 3:** Physiological and biochemical characteristics of endophytic bacteria CHR2-1.

Tests	Results	Tests	Results
Catalase	+	Glucose utilization	+
Lecithinase	−	Sucrose utilization	+
Contact enzyme	+	Xylose utilization	+
Urease	−	Fructose utilization	+
Aerobic type	+	Lactose utilization	+
Growth at pH 5.7	+	Maltose utilization	+
Indole acetic acid	−	Galactose utilization	+
Litmus milk reaction	−	D-Glucose fermentation	Produce sour
Phenylalanine oxidative deamination	−	L-Arabinos fermentation	Produce sour
H_2_S production	−	D-Xylose fermentation	Produce sour
Glutin liquefaction	+	D-Mannit fermentation	Produce sour
Starch hydrolysis	+	2% NaCl	+
Methyl red	+	5% NaCl	+
VP reaction	+	7% NaCl	+
Propionate utilization	−		

“+” positive, “−” negative.

The full length of the 16S rDNA nucleotide sequence of endophytic bacteria CHR2-1 was 1,459 base pair (bp). The sequence was deposited in GenBank (NCBI) under accession JX502843. 16S rDNA sequence of the strain was analyzed by the online BLAST server of NCBI, and it was found that the strain showed higher homology with the *bacillus* genus (Figure 2). The construction of the phylogenetic tree displayed that the endophytic bacteria CHR2-1 was 100% similar to *B. subtilis* A17 (KC434971). Based on morphological, physiological, and biochemical characteristics, the CHR2-1 strain was identified as *B. subtilis*.

**Figure 3:**
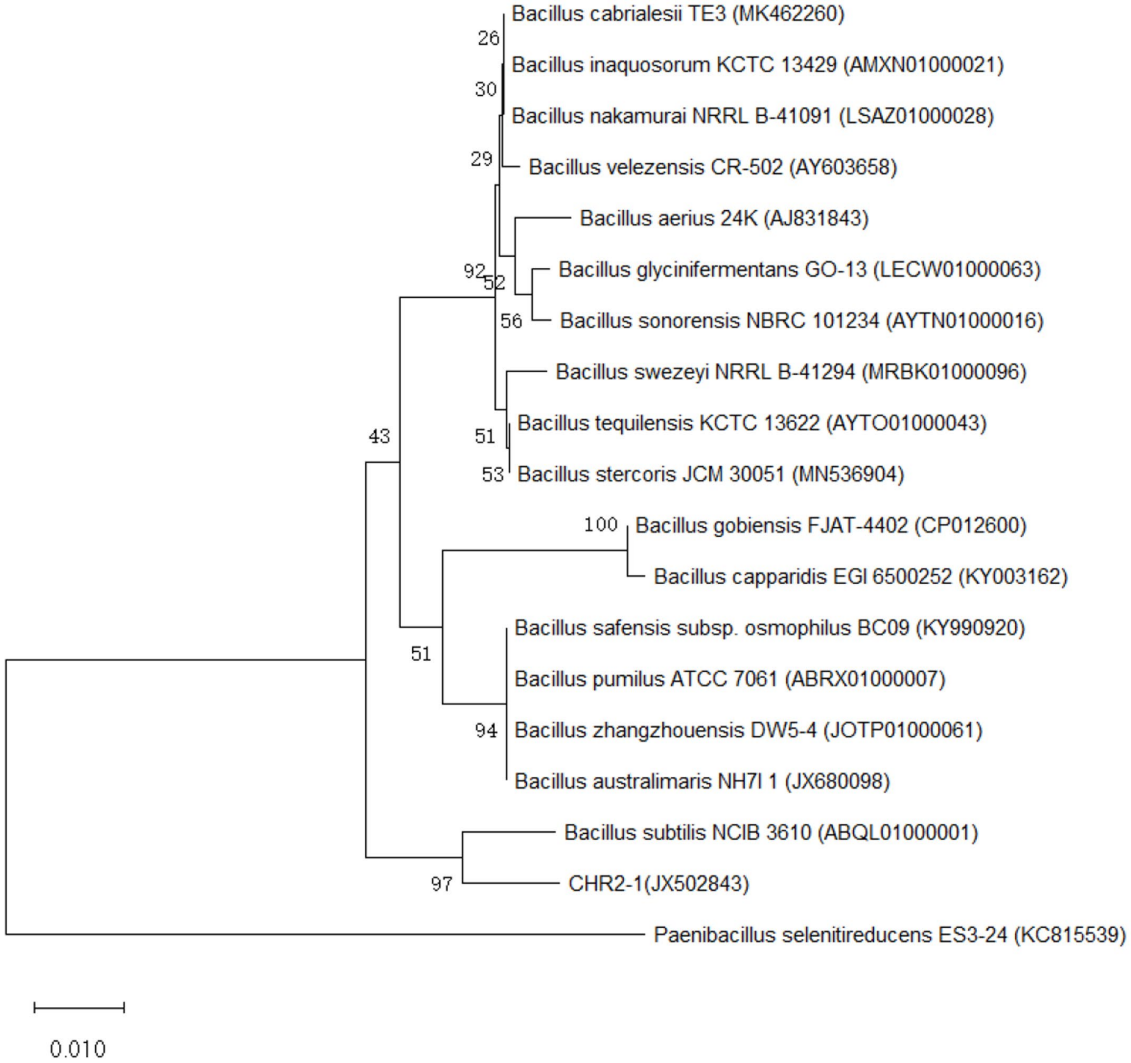
CHR2-1: Phylogenetic tree.

Neighbor-joining phylogenetic tree based on 16S rDNA sequence (1,459 bp), showing the phylogenetic relation between CHR2-1 strain (JX502843) and the other strains closely related to *B. subtilis* A17 (KC434971) (Figure 3). The numbers at nodes are percentages indicating the levels of bootstrap support, based on a neighbor-joining analysis of 1,000 resampled data sets. Only values of >50% are shown.

### 3.3 The effect of endophytic bacteria CHR2-1 on spore germination

Using the spore germination method, the filtrate from the LB and NA culture solution of the endophytic bacteria CHR2-1 was assayed to inhibit the spore germination of 5 kinds of plant pathogenic fungi, and the results are shown in Table 4.

**Table 4:** The effect of CHR2-1 on spore germination of the five target fungi.

Target fungi	Inhibiting rate (%)	
	Filtrate from LB	Filtrate from NA
FO (ATCC 76255)	59.21 Bb	48.05 Aa
FO (ATCC 18467)	77.09 Aa	42.40 Aa
CM (ATCC 96167)	60.24 Bb	14.67 Cc
CG (ATCC 16330)	31.90 Cc	25.70 Bb
PP (ATCC 22386)	23.03 Dd	21.06 Bb

Table 4 shows that the filtrate of the LB and NA culture solutions of the endophytic bacteria CHR2-1 displayed strong inhibitory effects on spore germination of the five target fungal species. Among them, the LB culture solution filtrate showed the strongest inhibitory effect on spore germination of FO (ATCC 18467), with an inhibitory rate of 77.09%. Second, the filtrate also exhibited strong inhibitory effects on spore germination of CM (ATCC 96167) and FO (ATCC 76255). In contrast, the inhibiting activity of the NA culture solution filtrate on the spore germination of target fungi was not satisfactory; the inhibiting rates were lower than 50%. Moreover, it could be found that the different culture solutions of the endophytic bacteria CHR2-1 showed different inhibiting activity, which implied that it was important to select a suitable culture solution to incubate the endophytic bacteria CHR2-1.

### 3.4 Results of the pot experiment of endophytic bacteria CHR2-1 against banana wilt disease

**Figure 4:**
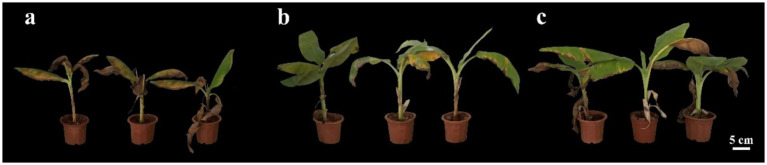
Potted plant fruiting diagram. **(a)** Sterile water treatment group, **(b)** CHR2-1 treatment group, **(c)** 95% hymexazol DP.

Control effect was listed in Table 5 to apply endophytic bacteria CHR2-1 against FO (ATCC 76255) *in vitro*. After 14 days of inoculating the pathogen, all treatment groups displayed the disease symptoms; the disease index of the sterile water treatment group was 38.33%, and the disease index of the CHR2-1 strain treatment group was lower (8.12%). With the extension of culture time, the disease index increased. After 30 days of inoculation, the disease index of the sterile water treatment group was 78.64%, but the disease index of the CHR2-1 strain and 95% hymexaxzol DP treatment groups developed slowly, and the control effect of the CHR2-1 strain reached 65.62%, which was not a significant difference with 95% hymexaxzol DP treatment (61.00%). Thus, the endophytic bacteria CHR2-1 can effectively prevent and control banana wilt disease (Figure 4).

**Table 5:** Control effect of the CHR2-1 strain against banana wilt disease.

Treatment	Disease index (%)			Control effect (%)
	14 days	22 days	30 days	
Sterile water	38.33	51.67	78.64	–
CHR2-1	8.12	16.51	27.04	65.62 Aa
95% Hymexaxzol DP	11.63	22.14	30.67	61.00 Aa

### 3.5 Growth-promoting effect of endophytic bacteria CHR2-1 on banana seedlings

In a pot experiment, the growth-promoting effect of endophytic bacteria CHR2-1 on banana seedlings was tested. From Table 6, the increment of plant height (5.25 cm) and stem diameter (0.66 cm) showed a significant difference between the CHR2-1 strain and LB culture solution or sterile water. At the same time, the fresh weight of banana seedlings had a significant increase after using the CHR2-1 strain; the values of the overground part and underground part were 156.16 and 23.45 g, respectively, which was a significant difference from LB culture solution or sterile water. Thus, the endophytic bacteria CHR2-1 could promote banana growth.

**Table 6:** Growth-promoting effect of the CHR2-1 strain.

Treatment	Increment (cm)		Fresh weight (g)	
	Plant height	Stem diameter	Overground part	Underground part
CHR2-1	5.25Aa	0.66 Aa	156.16 Aa	23.45 Aa
LB	3.34 Bb	0.41 Bb	132.47 Bb	16.76 Bb
Sterile water	3.73 Bb	0.49 Bb	128.45 Bb	17.23 Bb

### 3.6 Optimized results of the culture conditions of endophytic bacteria CHR2-1

#### 3.6.1 Culture temperature

**Figure 5:**
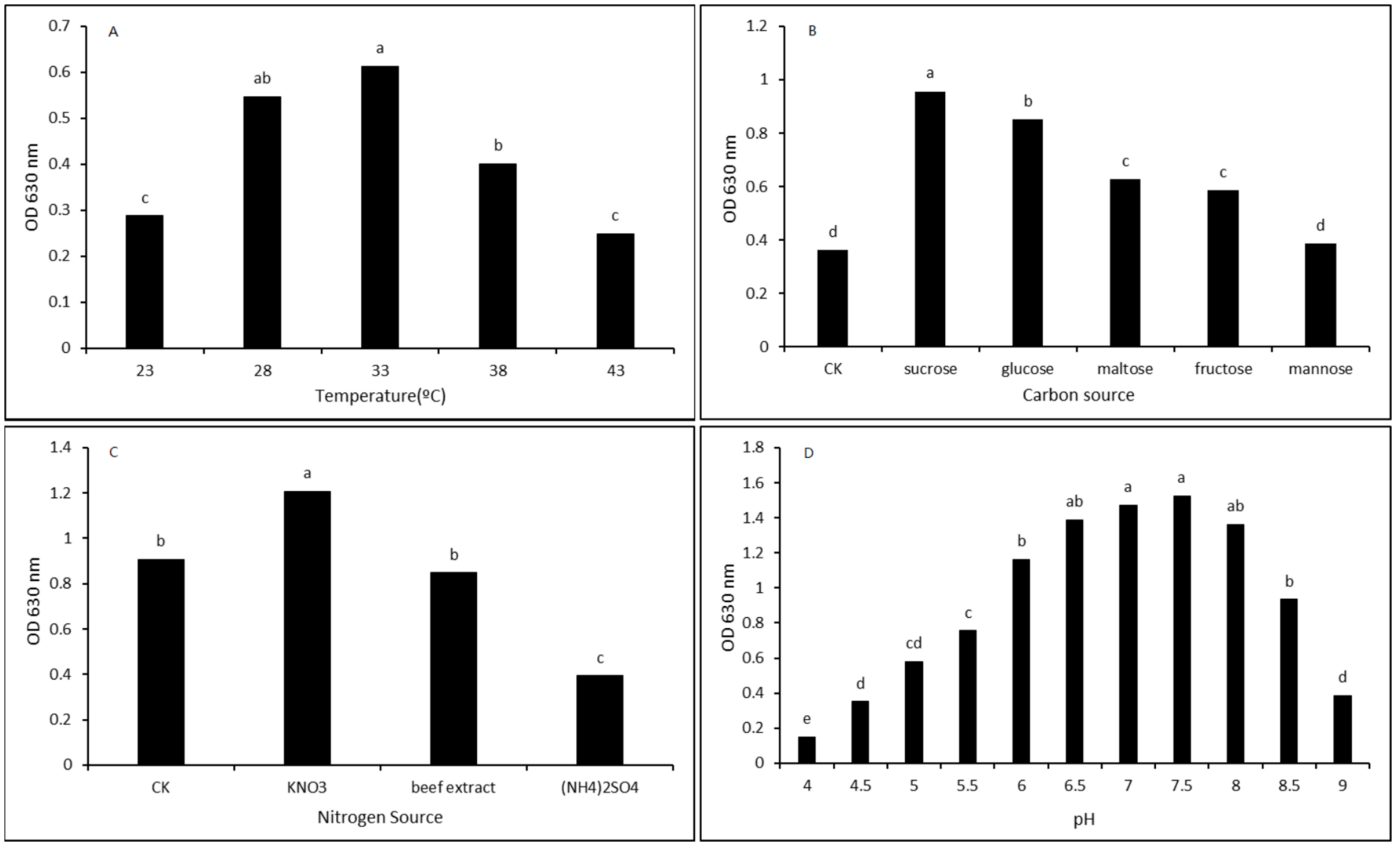
The effect of culture conditions of the CHR2-1 strain on growth. **(A)** Temperature; **(B)** Carbon sources; **(C)** Nitrogen sources; **(D)** The pH value.

Inoculated the CHR2-1 strain in LB culture solution to cultivate for 24 h; from Figure 5A, it was found that temperature was an important factor that impacted on the growth of the strain. When the temperature was 23 °C, the OD value was merely 0.288; as the temperature increased, the OD value became bigger, and then decreased. When the temperature reached 33 °C, the growth amount of the strain reached its maximum, with an OD value of 0.613, which was not a significant difference from the temperature of 28 °C. Thus, the suitable temperature range for cultivating the strain was 28–33 °C.

#### 3.6.2 Carbon source

Based on the optimized temperature results, the temperature of 33 °C was selected to cultivate the strain to optimize the carbon source, including sucrose, glucose, maltose, fructose, and mannose (Figure 5B). The additional carbon source could promote the growth of the strain; the effect of sucrose was the best, and the OD value was 0.956, which was a significant difference from other carbon sources. Second, the effects of glucose, maltose, and fructose were also excellent, with a significant difference compared to the control. Thus, sucrose was selected as a suitable carbon source to cultivate the strain.

#### 3.6.3 Nitrogen source

On the basis of the optimized temperature and carbon source culture solution, the yeast powder in the culture solution was substituted for KNO_3_, (NH_4_)_2_SO_4,_ and beef extract (Figure 5C). KNO_3_ was the best of all the nitrogen sources, and the OD value was 1.206, which was significantly different from the other nitrogen sources. The beef extract was the same as the control yeast powder, without significant differences. Thus, KNO_3_ was a suitable nitrogen source for this strain.

#### 3.6.4 The pH value

Adjusting the pH values on the basis of optimized carbon source and nitrogen source, the results showed that the pH played an important role in the growth amount of the strain. When pH value was 4.0, the OD value of the strain is only 0.151, then, with the pH value increasing, the growth amount of the strain tended to increase at first, the OD value reached its maximum of 1.525, when pH was 7.5, and then the growth amount decreased, when pH value was 9.0, the OD value was only 0.387. It was noteworthy that the OD value (1.472) had no significant difference between the pH 7.0 and pH 7.5. Thus, the suitable pH value for cultivating the strain was 7.0 ~ 7.5 (Figure 5D).

### 3.7 Optimized results of fermentation conditions of endophytic bacteria CHR2-1

#### 3.7.1 The establishment of a mathematical model and the significance test

Take the peptone, potassium nitrate, sodium chloride, and sucrose as experimental factors, adopting the quadratic general rotary unitized design to optimize the formula of CHR2-1 fermentation culture medium; the test design and the results are shown in Table 7 and the analysis of variance is shown in Table 8.

**Table 7:** Secondary universal rotation combination design and results.

Processing	*x*_1_ (Peptone) g/L	*x*_2_ (Sucrose) g/L	*x*_3_ (Potassium nitrate) g/L	*x*_4_ (Sodium chloride) g/L	Inhibition zone (mm)
1	1	2.75	2.75	5.5	22.48
2	3.25	1.625	1.625	3.25	10.28
3	3.25	3.875	1.625	3.25	17.18
4	3.25	1.625	3.875	3.25	16.93
5	3.25	3.875	3.875	3.25	35.37
6	3.25	1.625	1.625	7.75	19.44
7	3.25	3.875	1.625	7.75	17.06
8	3.25	1.625	3.875	7.75	8.2
9	3.25	3.875	3.875	7.75	17.04
10	5.5	0.5	2.75	5.5	11.22
11	5.5	5	2.75	5.5	18.64
12	5.5	2.75	0.5	5.5	8.18
13	5.5	2.75	5	5.5	11.47
14	5.5	2.75	2.75	1	7.99
15	5.5	2.75	2.75	10	18.19
16	5.5	2.75	2.75	5.5	9.65
17	5.5	2.75	2.75	5.5	9.64
18	5.5	2.75	2.75	5.5	8.92
19	5.5	2.75	2.75	5.5	11.48
20	5.5	2.75	2.75	5.5	9.39
21	5.5	2.75	2.75	5.5	9.36
22	5.5	2.75	2.75	5.5	9.04
23	7.75	1.625	1.625	3.25	10.87
24	7.75	3.875	1.625	3.25	11.95
25	7.75	1.625	3.875	3.25	9.37
26	7.75	3.875	3.875	3.25	18.99
27	7.75	1.625	1.625	7.75	22.7
28	7.75	3.875	1.625	7.75	11.71
29	7.75	1.625	3.875	7.75	15.75
30	7.75	3.875	3.875	7.75	14.53
31	10	2.75	2.75	5.5	18.42

**Table 8:** Analysis of variance table of test results.

Source of variation	Quadratic sum	Free degree	Mean square	Partial correlation	*F*-value	*Q*-value	Conspicuousness
*x*_1_	129.0052	1	122.3492	0.6808	14.2693	0.0023	**
*x*_2_	91.5851	1	89.0076	−0.6155	9.9889	0.0074	**
*x*_3_	16.1039	1	16.2314	−0.3251	1.9114	0.2122	
*x*_4_	9.1901	1	9.7719	−0.265	1.0628	0.3642	
*x*_1_^2^	129.2994	1	124.6085	0.7025	13.7864	0.0024	**
*x*_2_^2^	90.2707	1	85.4848	0.6281	9.6479	0.0085	**
*x*_3_^2^	6.4868	1	6.3303	0.2067	0.7383	0.4383	
*x*_4_^2^	47.1065	1	47.1065	0.4974	5.4479	0.0416	*
*x*_1_ *x*_2_	139.3256	1	138.1257	−0.7366	15.1503	0.0016	**
*x*_1_ *x*_3_	9.8431	1	10.6220	−0.2666	1.0082	0.3377	
*x*_1_ *x*_4_	68.1225	1	64.6883	0.599	7.0953	0.0194	*
*x*_2_ *x*_3_	111.7256	1	117.1405	0.7163	13.2243	0.0034	**
*x*_2_ *x*_4_	101.4651	1	106.3827	−0.6454	11.1291	0.0047	**
*x*_3_ *x*_4_	73.5621	1	72.2845	−0.5819	7.8621	0.0164	*
Regress	984.7224	15	65.6482	*F* = 6.8905	*F*_0.01 (15, 16)_ = 3.41		
Remainder	152.4371	16	9.5273				
Misfit	26.8178	10	2.6818	*F*_Lf_ = 2.5974	*F*_0.05 (10, 6)_ = 4.06		
Error	6.1951	6	1.0325				
Total	1170.172	32					

**p < 0.01, very significant; *p < 0.05, significant.

Conduct the fitting of the acquired test data using the Series 13.0 data processing system (DPS), calculating each regression coefficient to obtain the regression model for the inhibition zone with the four factors: peptone, sucrose, potassium nitrate, and sodium chloride.


$$\begin{array}{c} y=9.72944+2.24553x_{1}-1.89203x_{2}-0.81468x_{3}\\ -0.61543x_{4}-2.05089\times 12-1.69869{x_{2}}^{2}\\ -0.46794{x_{3}}^{2}-1.27648{x_{4}}^{2}-6.05002x_{1}x_{2}\\ -5.08007x_{1}x_{3}+1.97548x_{1}x_{4}+2.62809x_{2}x_{3}\\ -2.50451x_{2}x_{4}-2.88667x_{3}x_{4} \end{array}$$ (1)

Where y, Diameter of inhibition zone, the predicted response value of the regression model, usually in millimeters; x_1_, Content of peptone in the fermentation medium (unit: g/L); x_2_, Content of sucrose in the fermentation medium (unit: g/L); x_3_, Content of potassium nitrate in the fermentation medium (unit: g/L); x_4_, Content of sodium chloride in the fermentation medium (unit: g/L); Constant term 9.72944, The predicted basic diameter of inhibition zone when the coded values of all factors are 0; Linear coefficient 2.24553: Represents the main effect of a single factor on the inhibition zone; Quadratic coefficient –2.05089: Represents the nonlinear effect of the factor on the response; Interaction coefficient –6.05002: Represents the interaction effect between two factors.

It is found through variance analysis based on the test results that (Table 9): the quadratic partial regression coefficient of peptone and sucrose has reached the extremely significant level (*p* < 0.01), the quadratic partial regression coefficient of sodium chloride reaches the significant level (*p* < 0.05), indicating that the various influencing factors in test are not in a simple linear relationship. Moreover, from the regression equation test for lack of fit, *F*_Lf_ = 2.5974 < *F*_0.05_ (10, 6) = 4.06, and the regression equation fitting test *F* = 6.8905 > *F*_0.01_ (15, 16) = 3.41, it can be seen that the insufficiency of the lack of fit of the equation is not very significant. In contrast, the equation regression is extremely significant; therefore, the fitting of this regression model is good, and the model is established. By removing the insignificant items under the level of α = 0.10 from the model, the regression equation is simplified as:


$$\begin{array}{c} y=9.72944+2.24553x_{1}-1.89203x_{2}-2.05089{x_{1}}^{2}\\ -1.69869{x_{2}}^{2}-1.27648{x_{4}}^{2}-6.05002x_{1}x_{2}\\ +1.97548x_{1}x_{4}+2.62809x_{2}x_{3}\\ -2.50451x_{2}x_{4}-2.88667x_{3}x_{4} \end{array}$$ (2)

Where y, Diameter of inhibition zone, the predicted response value of the regression model, usually in millimeters; x_1_, Content of peptone in the fermentation medium (unit: g/L); x_2_, Content of sucrose in the fermentation medium (unit: g/L); x_3_, Content of potassium nitrate in the fermentation medium (unit: g/L); x_4_, Content of sodium chloride in the fermentation medium (unit: g/L); Constant term 9.72944: The predicted basic diameter of inhibition zone when the coded values of all factors are 0; Linear coefficient 2.24553: Represents the main effect of a single factor on the inhibition zone; Quadratic coefficient –2.05089: Represents the nonlinear effect of the factor on the response; Interaction coefficient –6.05002: Represents the interaction effect between two factors.

**Table 9:** Partial regression sub-model for each test factor and antibacterial effect.

Test factor	Code	Regression model
Peptone	*x*_1_	*y*_1_ = 9.72944+2.24553*x*_1_−2.05089*x*_1_^2^
Sucrose	*x*_2_	*y*_2_ = 9.72944−1.89203*x*_2_−1.69869*x*_2_^2^
Potassium nitrate	*x*_3_	*y*_3_ = 9.72944−0.81468*x*_3_−0.46794*x*_3_^2^
Sodium chloride	*x*_4_	*y*_4_ = 9.72944−0.61543*x*_4_−1.27648*x*_4_^2^

##### 3.7.1.1 Analysis for the main effect of the mathematical model

As the regression model itself has gone through dimensionless coding substitution, its partial regression coefficient has been standardized, and there is no any correlation between its monomial regression coefficients or between the monomial regression coefficients and the regression coefficients of the interaction terms and the quadratic terms, therefore, the relative importance of each factor to the target function can be determined by the absolute value of the monomial regression coefficients in the mathematical model. In this mathematical model, the order of the number of absolute values of each monomial regression coefficient is *x*_1_ > *x*_2_ > *x*_3_ > *x*_4_; therefore, among all the test factors, peptone has the greatest impact on the antibacterial effect, followed by sucrose, potassium nitrate, and the impact of sodium chloride is the smallest.

#### 3.7.2 Effect analysis for a single factor of the mathematical model

Use the dimension reduction method to fix any three variables of the mathematical model at the level of 0, to obtain the partial regression model of any of the four variables (see Table 9), and the sub-model is equivalent to the single factor test under a specific condition.

**Figure 6:**
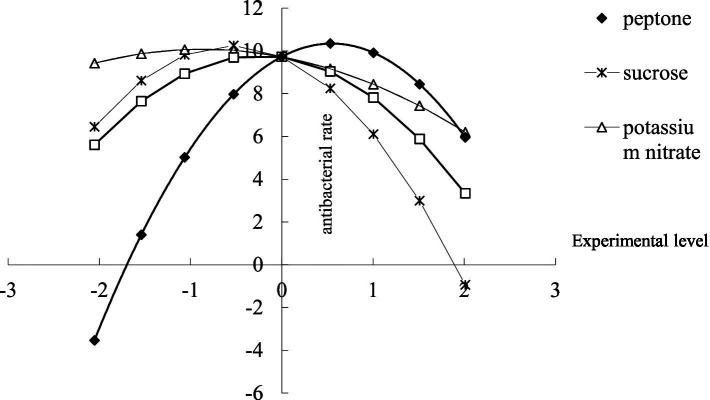
Analysis of single-factorial effects.

Substitute the level value of each factor into the sub-model stated above to obtain the effect figure of the factor on the yield (Figure 6).

It can be seen from Figure 6 that, in the range of the test, each test factor has an upward parabolic relationship with the yield. Take the peptone as an example, peptone increases from the −2.022 level (the additive amount is 0.475 g/L) to 0.518 level (the additive amount is 3.33 g/L), and the inhibition zone increases from 0 to 10.34 mm. When the additive level exceeds 0.518, the bacteriostatic rate starts to decrease, and when the additive level is 2.022 (the additive amount is 5.025 g/L), the inhibition zone decreases to 5.96 mm. Similarly, the highest bacteriostatic effect is achieved when the sucrose is at the level of −0.5095 (the additive amount is 2.177 g/L), and the size of the inhibition zone is 10.25 mm; the highest bacteriostatic effect is achieved when potassium nitrate is at the level of −1.066 (the additive amount is1.55 g/L), and the size of the inhibition zone 10.06 mm; the highest bacteriostatic effect is achieved when sodium chloride is at the level of 0 (the additive amount is 5.500 g/L), and the size of the inhibition zone is 9.729 mm.

#### 3.7.3 Analysis for the interaction effect of the mathematical model

Determine two factors among the peptone, sucrose, potassium nitrate, and sodium chloride at level 0, to, respectively, obtain the sub-models of the other two factors with the yield:


$$\begin{array}{c} y_{\left(1,2\right)}=9.72944+2.24553x_{1}-1.89203x_{2}-2.05089{x_{1}}^{2}\\ -1.69869{x_{2}}^{2}-6.05002x_{1}x_{2} \end{array}$$ (3)

$$\begin{array}{c} y_{\left(1,3\right)}=9.72944+2.24553x_{1}-0.81468x_{3}-2.05089{x_{1}}^{2}\\ -0.46794{x_{3}}^{2}-5.08007x_{1}x_{3} \end{array}$$ (4)

$$\begin{array}{c} y_{\left(1,4\right)}=9.72944+2.24553x_{1}-0.61543x_{4}\\ -2.05089{x_{1}}^{2}-1.27648{x_{4}}^{2}+1.97548x_{1}x_{4} \end{array}$$ (5)

$$\begin{array}{c} y_{\left(2,4\right)}=9.72944–1.89203x_{2}-0.61543x_{4}\\ -1.69869{x_{2}}^{2}-1.27648{x_{4}}^{2}-2.50451x_{2}x_{4} \end{array}$$ (6)

$$\begin{array}{c} y_{\left(2,3\right)}=9.72944–1.89203x_{2}-0.81468x_{3}\\ -1.69869{x_{2}}^{2}-0.46794{x_{3}}^{2}+2.62809x_{2}x_{3} \end{array}$$ (7)

$$\begin{array}{c} y_{\left(3,4\right)}=9.72944–0.81468x_{3}-0.61543x_{4}-0.01264{x_{3}}^{2}\\ -1.27648{x_{4}}^{2}-2.88667x_{3}x_{4} \end{array}$$ (8)

Where y(i,j), Predicted value of the inhibition zone diameter (mm) when factors x i and x j vary and all other factors are fixed at the central level; x_1_, x_2_, x_3_, x_4_, Coded variables for peptone, sucrose, potassium nitrate, and sodium chloride, respectively. Their conversion relationships with the actual concentrations are the same as previously described; Constant term, Derived from the full regression model, representing the basic response value at the central point; Linear and quadratic coefficients, Reflect the main effect and nonlinear effect of a single factor in the specific pairwise analysis. Interaction coefficient, Directly shows the strength and direction of the interaction between two factors. A positive coefficient indicates synergistic promotion, while a negative coefficient indicates synergistic inhibition.

**Figure 7:**
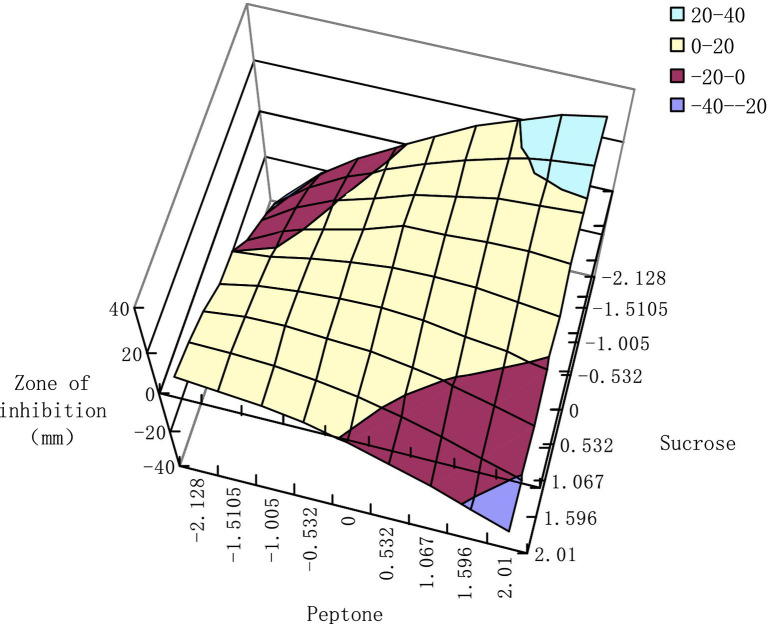
Protein and sucrose interaction effect response surface.

Among the six sub-models, the significance of (2) (*F*-value = 15.1503) is the best; therefore, the analysis of the interaction effect is done by taking the peptone and sucrose as examples. Substituting −2.128, −1.5105, −1.005, ± 0.532, 0, 1.067, 159.6, and 2.01, these nine levels into model (2), to get the interaction effect of the bacteriostasis effect produced on the bacterial strain by peptone and sucrose, to further draw the inhibition zone data into a response surface (Figure 7).

According to the extreme value of multivariate function theory, when the CHR2-1bacteriostasis effect reaches its maximum in the response surface, the levels of peptone and sucrose are, respectively, calculated as −0.8416 and 0.9517. It can be seen from the figure that the response surface is divided into four zones; the impact of the additive amount of the peptone and sucrose in each zone on the CHR2-1bacteriostasis effect is listed in Table 10.

**Table 10:** Impact of the peptone and sucrose dosage changes on the bacteriostasis effect of CHR2-1.

Zone	Factor changes		Change in bacteriostasis effect
*x*_1_ < −0.8416, *x*_2_ < 0.9517	Peptone↑	Sucrose↑	↑
*x*_1_ > −0.8416, *x*_2_ < 0.9517	Peptone↑	Sucrose -	↓
	Peptone -	Sucrose↑	↑
*x*_1_ < −0.8416, *x*_2_ > 0.9517	Peptone↑	Sucrose -	↑
	Peptone -	Sucrose↑	↓
*x*_1_ > −0.8416, *x*_2_ > 0.9517	Peptone↑	Sucrose↑	↓

“↑” indicates “increase,” “↓” indicates “decrease,” and “-” indicates “no change”.

Similarly, calculate the extreme value of the peptone and potassium nitrate, peptone and sodium chloride, sucrose and potassium nitrate, sucrose and sodium chloride, potassium nitrate and sodium chloride response surfaces, which, respectively, are: (−0.2846, 0.6718), (0.6876, 0.291), (1.0495, 2.0766), (−1.3699, 1.1029), (2.668, −1.847). According to the zoning of the extreme value response surface area, the variation trend of the CHR2-1 antibacterial effect under different levels of factors can be found directly.

#### 3.7.4 Model optimization and test verification

Analyze the relevant data acquired according DPS software, taking five levels −2.132, −1.518, 0, 1.066, and 2.188 for each factor, using computer to seek the optimization from 54 = 625 schemes, and it is obtained that when the protein peptone additive level is 2.188 (the amount of addition is 10.423 g/L), sucrose additive levels is −1.518 (the amount of addition is 1.042 g/L), potassium nitrate addition level is −1.066 (the amount of addition is 1.558 g/L), and the sodium chloride addition level is 0 (the amount of addition is 5.5 g/L), the bacteriostatic effect of bacterial strain CHR2-1 is the most obvious, and the inhibition zone reaches 40 mm. The test results were verified using the experimental design and optimization methods of the DPS software. The results showed that, under this addition level, the inhibition zone of bacterial strain CHR2-1 is 38.33 mm, close to the predicted value (40 mm), which indicates that the mathematical model established in this study has certain predictability.

## 4 Conclusion and discussion

### 4.1 Isolation and screening of endophytes and antagonistic activity of CHR2-1

Eight endophytic fungi and 15 endophytic bacteria were isolated from the roots, stems, and leaves of Hainan *C. hainanensis* by surface sterilization. Nine tropical pathogenic fungi were used as targets, and only six endophytic bacteria exhibited antibacterial activity using the streak plate method. Among them, CHR2-1, isolated from the roots, had the greatest effect. This strain had a broad antibacterial spectrum, with inhibition rates of over 50% against nine target fungi, including *F. oxysporum* f. sp. cubense and *R. solani*. The inhibition rate against *F. oxysporum* f. sp. cubense was the highest (88.40%), and the filtrate of its LB culture medium could inhibit the spore germination of this fungus by nearly 60%. Its biocontrol mechanism may be achieved through the following pathways in a coordinated manner: Competitive exclusion: *B. subtilis* can quickly colonize the rhizosphere, surface, or interior of plants, effectively preventing the reproduction of pathogenic microorganisms through nutrient competition and spatial competition, and interfering with the infection of plant pathogenic microorganisms on plants (Pomerleau et al., 2024). Secretion of antibacterial substances: *B. subtilis* can produce various antibacterial substances, such as lipopeptide antibiotics (such as surfactin), chitinase, protease, etc. For example, recent studies have revealed that *Bacillus amyloliquefaciens* TG1-2 can significantly inhibit protein accumulation of the NatA acetyltransferase complex in *Verticillium dahliae* by producing surfactin, thereby affecting its protein acetylation homeostasis and ultimately leading to fungal cell apoptosis (Zhang et al., 2025). The filtrate of the LB culture medium of CHR2-1 can effectively inhibit the spore germination of *F. oxysporum* f. sp. cubense (inhibition rate nearly 60%), suggesting that it may secrete similar small-molecule antibacterial substances or cell-wall-degrading enzymes. Lysing action: *B. subtilis* can adhere to the hyphae of pathogenic fungi and produce lysing substances during growth, causing the hyphae to break, disintegrate, or dissolve the cytoplasm (Sansinenea et al., 2016). Induction of systemic resistance: *B. subtilis* can also induce the plant’s own defense system and enhance the plant’s resistance to pathogenic fungi.

### 4.2 Disease control and growth-promotion effects of CHR2-1 on potted banana seedlings

Potted experiments demonstrated that CHR2-1 could delay the disease progression of banana seedlings, achieving a 65.62% control efficacy within 30 days (comparable to metalaxyl), and significantly enhance growth indicators such as plant height, stem diameter, and fresh weight. Based on *in vitro* and *in vivo* test results, it is speculated that its biocontrol mechanism may involve the secretion of secondary metabolites to inhibit pathogens and the production of plant hormones to promote growth, indicating potential for development as a biocontrol agent (Sarkar et al., 2025). However, the specific mechanism remains to be further studied. Current research indicates that successful colonization by endophytes is a key step for their biocontrol function. Studies have shown that certain endophytic bacteria (such as *B. subtilis* BS21 and BS22) can not only colonize their natural host plants but also various non-natural host plants (Kuramshina et al., 2023). The effective colonization of CHR2-1 in the rhizosphere and within banana seedlings may enhance the seedlings’ resistance to Fusarium wilt disease through continuous secretion of antibacterial substances and their migration in the vascular bundles, providing systemic protection. Additionally, after colonization, endophytes often stimulate the activity of plant defense enzymes [such as polyphenol oxidase (PPO), peroxidase (POD), and phenylalanine ammonia lyase (PAL)], thereby enhancing the immune level of the host plants (Wang et al., 2024).

### 4.3 Optimization of CHR2-1 fermentation conditions

Single-factor experiments determined the optimal growth conditions for CHR2-1: LB medium supplemented with 10 g/L sucrose (carbon source) and 5 g/L KNO₃ (nitrogen source), pH 7.5, 33 °C, shaken at 180 rpm for 24 h, and the strain grows well between 28 and 33 °C. Based on this, a second-order central composite design was used to establish a four-factor (peptone, potassium nitrate, sodium chloride, sucrose) mathematical model, resulting in the optimized culture medium. Under these conditions, the inhibition zone against *F. oxysporum* f. sp. cubense reached 40 mm, and the model prediction was accurate. Analysis of the main effects showed the order of influence on antibacterial activity as: peptone > sucrose > potassium nitrate > sodium chloride (with peptone and sucrose having extremely significant effects); the interaction effects among factors were complex, and the optimal formulation was not a simple accumulation of individual optimal values. Fermentation factors such as aeration and agitation speed require further study. The fermentation medium optimized through the second-order central composite design significantly enhanced the antibacterial activity of CHR2-1. This optimization strategy has profound metabolic regulatory implications:

Core role of peptone: As an organic nitrogen source, peptone exhibited a highly significant partial regression coefficient, profoundly affecting antibacterial activity. This may be because peptone is rich in amino acids and small peptides, which can promote *B. subtilis* to synthesize antibacterial lipopeptides (such as surfactin and iturin), whose structural units are directly derived from amino acid metabolism (Zhao et al., 2025).

Carbon-nitrogen balance and metabolic flux reprogramming: The interaction between sucrose and peptone was highly significant, indicating that the carbon-to-nitrogen ratio plays a key regulatory role in the synthesis of secondary metabolites. An appropriate carbon-to-nitrogen ratio may facilitate the transition from the growth phase to the sporulation phase and activate quorum-sensing systems, thereby promoting efficient synthesis of antibacterial compounds (Romero-Rodriguez et al., 2018).

Synergistic effects of inorganic ions: In addition to maintaining osmotic pressure, NaCl and KNO₃ may serve as enzyme cofactors or signaling molecules, affecting the intracellular redox state and indirectly regulating the expression of antibacterial gene clusters (Zeng et al., 2015).

### 4.4 CHR2-1 identification

CHR2-1 is Gram-positive, with typical *B. subtilis* morphology and physiological and biochemical characteristics, including central spores and peritrichous flagella; 16S rDNA sequence analysis shows a 97% homology with *B. subtilis* NCIB 3610, and it was identified as *B. subtilis*. As an important biocontrol bacterium, *B. subtilis* has advantages such as a broad-spectrum antibacterial activity, diverse mechanisms of action, ease of survival and colonization in the environment, simple production, processing, and transportation, convenient application, and long shelf life. Strain CHR2-1 was identified as *B. subtilis* and has the potential to be developed into an ideal biocontrol agent. Its potential development lies not only in its excellent control effect against banana wilt (65.62%) but also in its dual function of “disease prevention and growth promotion.” This dual function may stem from its ability to secrete plant hormones (such as IAA) and siderophores, which promote growth. Combined with *B. subtilis*’s broad antibacterial spectrum (Grobelak and Hiller, 2017), easy survival and colonization, and ease of production and storage, this strain is expected to be developed into an ideal biocontrol agent.

## 5 Summary and outlook

The biocontrol effect of CHR2-1 (*B. subtilis*) stems from multiple mechanisms, including competition, lytic activity, production of antimicrobial substances, and induction of plant resistance. Successful colonization of the strain is crucial for exerting its biocontrol effect, while optimizing fermentation conditions can effectively increase the yield of its secondary metabolites. Future research can further focus on the isolation, purification, and structural identification of CHR2-1’s antimicrobial substances, as well as improving the strain through genetic engineering to enhance its biocontrol efficacy.

## Data Availability

The data presented in this study are publicly available. The data can be found here: https://www.ncbi.nlm.nih.gov/genbank, accession JX502843.
